# Prevalence of Perinatal Deaths in a Tertiary Care Hospital of Nepal

**DOI:** 10.31729/jnma.4431

**Published:** 2019-06-30

**Authors:** Yam Prasad Dwa, Sunita Bhandari

**Affiliations:** 1 Department of Obstetrics & Gynecology, KIST Medical College & Teaching Hospital, Imadol, Lalitpur, Nepal

**Keywords:** *live births*, *perinatal mortality*, *pregnancy*

## Abstract

**Introduction:**

Perinatal mortality indicates quality of maternal and neonatal care and is high in Nepal. This study was conducted to find out the prevalence of perinatal deaths in a tertiary care center.

**Methods:**

This descriptive cross-sectional study was conducted from July 2017 to June 2018 at KIST Medical College and Teaching Hospital. Details of each perinatal death were filled in predesigned proforma from hospital in-patient records within 24 hours of perinatal death. The total of 1088 cases were selected for the study and convenience sampling was done. Statistical analysis was done with Statistical Package for Social Sciences version 17.0.

**Results:**

Prevalence of perinatal death is 16 (1.46%) out of 1095 births. In the same way, perinatal mortality rate, stillbirth rate and early neonatal mortality rate were calculated and found to be 14.61 per 1000 births, 8.21 per 1000 births and 6.44 per 1000 live births respectively. Preterm neonates constituted of 71.4% of early neonatal deaths.

**Conclusions:**

Perinatal mortality rate was 50% lower than that of national survey, however comparable with study at another tertiary care center at Kathmandu. Stillbirth and prematurity contributed significantly to perinatal mortality. Provision of good antenatal surveillance, identification of high risk pregnancies and good neonatal care to preterm neonates would be required to reduce perinatal mortality.

## INTRODUCTION

Perinatal mortality represents the quality of the maternal and neonatal care, as well as the socioeconomic status of the country. World Health Organization (WHO) estimates that three million babies die in early neonatal period while 2.6 million are stillborn per year worldwide, of which one in three deaths occur during delivery which can be largely prevented.^1^ Though, the perinatal mortality rate has decreased from 45 per 1000 in 2006 to 31 per 1000 births in 2016,^2^ perinatal mortality in Nepal continues to be one of the major challenge.

Analysis of perinatal deaths will enable clinicians and policy makers to determine magnitude and identify areas of improvement, thus to formulate plan accordingly to reduce perinatal deaths in future.

This study was conducted with the aims of determining the prevalence of perinatal deaths in at KIST Medical College and Teaching Hospital.

## METHODS

This was a hospital based descriptive cross-sectional study conducted from July 2017 to June 2018 at KIST Medical College and Teaching Hospital. An ethical approval was obtained from institutional review committee prior to the study with Institutional review number 0127/074/075. All stillbirths (from 22 weeks of gestation or baby weighing 500 grams or more) and neonatal deaths occurring within first seven days of age during the study period were included. Babies born before 22 weeks of gestation or below 500 grams at birth were excluded from the study. Similarly, neonatal deaths after first seven days of age were also excluded. Convenience sampling was done and sample size was calculated using the formula,


$$\text{n}={\text{Z}}^{2}\times (\text{p}\times \text{q})/{\text{d}}^{2}$$

where,

n = sample size
p = prevalence, 50%
q = 1-p
d = margin of error, 3%
Z = 1.96 at 95% CI

Non-respondent rate of 2% was taken. Calculated sample size was 1088. Total sample size taken was 1095. Selection and information bias was minimized as possible by collecting data in the appropriately predesigned proforma. Statistical analysis was done with Statistical Package for Social Sciences (SPSS 17.0), point estimate at 95% CI was calculated along with frequency and proportion for binary data and analysis was done.

Details of each perinatal death were filled by the investigators within 24 hours of perinatal death from the hospital in-patient records in predesigned proforma. Details of maternal age, parity, gestational age, place and number of antenatal checkups, mode of delivery, weight of baby, perinatal outcome (stillbirths / early neonatal death) were recorded.

Perinatal mortality rate (PMR) was calculated as the total number of stillbirths (from 22 weeks of gestation or baby weighing at least 500 grams) plus early neonatal deaths (within 7 days of birth) per 1000 births. Similarly, Early Neonatal Mortality Rate (ENMR) was calculated as the deaths of neonates within 7 days of life per 1000 live births and stillbirth rate (SBR) was calculated as total number of fetal deaths per 1000 births.

## RESULTS

The total number of deliveries were 1088 including seven set of twins giving rise to toal births of 1095 during one year period. Prevalence of perinatal deaths is found to be 1.46% at 95% confidence interval. There were 16 cases of perinatal deaths with PMR of 14.61 per 1000 births. There were 9 stillbirths (SB) with stillbirth rate of 8.21 per 1000 births and 7 early neonatal deaths with ENND rate of 6.44 per 1000 live births.

Out of total perinatal deaths, 9 (56.25%) of mothers were in age group of 24-34 years while 6 (37.5%) were in age below 19 years and 1 (6.25%) in age group more than 35 years. Nine (56.25%) of total perinatal death occurred in multigravida mothers and 7 (43.75%) in primigravida. Eleven (68.75 %) of total perinatal deaths were delivered by vaginal delivery and 5 (31.25%) by lower segment cesarean section. Among them, 8 (50%) were male and 8 (50%) were female. About 13 (81.25%) of perinatal deaths were preterm births (22 -36 weeks) while 3 (18.75%) occurred in term pregnancy (Table 2).

**Table 1.** Deliveries and perinatal mortality.

Total deliveries	1088
Total births	1095
Twin births	7 (0.63%)
Total live births	1086
Perinatal mortality rate	14.61 per 1000 births
Stillbirth rate	8.21 per 1000 births
Fresh stillbirth rate	4.56 per 1000 births
Macerated stillbirth rate	3.65 per 1000 births
Early neonatal mortality rate	6.44 per 1000 live births
LSCS out of total deliveries	378 (35.75%)
Vaginal deliveries out of total deliveries	710 (65.25%)

**Table 2.** Gestational age of perinatal death.

Gestational age (weeks)	Still birth n	Early neonatal death n	Total perinatal death n (%)
<28	3	2	5 (31.25)
28-33	3	2	5 (31.25)
34-36	2	1	3 (18.75)
37-41	1	2	3 (18.75)
Total	9	7	16 (100)

## DISCUSSION

Globally perinatal deaths have reduced over the years, however the majority of deaths are occurring in south Asian and sub-Saharan countries.^3^ Similarly, global still birth rate was shown to reduce from 24.7 per 1000 births in 2000 to 18.4 per 1000 births in 2015, with slowest decline in sub-Saharan and south Asian countries.^1^ Perinatal mortality thus reflects quality of care of mothers during antenatal period, availability of medical care facilities, obstetric care during delivery and neonatal care facilities.

Similar to global trend perinatal mortality in Nepal too has reduced over the years. National demographic health surveys have demonstrated serial reduction in perinatal mortality; 45 per 1000 births in 2006, 38 per 1000 births in 2011 and 31 per 1000 births in 2016.^2^

Similarly neonatal mortality rate is also on improving trend as 33 per 1000 births to 21 per 1000 live births in 2016.^2^ This study demonstrated the PMR of 14.61 per 1000 births with SBR of 8.21 per 1000 births and ENMR of 6.44 per 1000 live births which is lower than the national data. This study was conducted at a tertiary care center in the Kathmandu valley where patients were from lower to middle socioeconomic strata, regular antenatal check-ups, labor monitoring as well as deliveries attended by trained nursing personnel, Obstetricians and Pediatricians. Availability of better resources in the obstetric and neonatal care and sample bias may the possible reasons for lesser perinatal deaths as compared to national data. This is also demonstrated in other tertiary care hospital based studies from Nepal.^4,5^

In a 2-year analysis of hospital records at Nepal Medical College Teaching Hospital, perinatal mortality rate was 29.4 per 100 births, SBR of 24.8 per 1000 births and ENMR of 4.7 per 1000 live births.^6^ A 2-year study in 2006 showed perinatal mortality of 21 per 1000 births and ENMR of 6.7 per 1000 live births at Kathmandu Medical College.^4^ Similarly a 13 year analysis of trends in perinatal mortality at Tribhuvan University Teaching Hospital (TUTH) showed reduction of perinatal deaths from 30 to 18 per 1000 births. There was significant decline in stillbirths but no considerable changes in early neonatal deaths over the study period.^5^ The perinatal mortality rate, SBR and ENMR of our study were comparable with this study.^5^

However, there is higher PMR in the community and hospitals outside valley in Nepal. At Dhanusha district, south-eastern region of Nepal, the perinatal mortality rate was 60 per 1000 births with stillbirth rate of 31 per 1000 births and the neonatal mortality rate 38 per 1000 live births.^7^ Similarly an one year study conducted at Manipal Medical College Teaching Hospital at Pokhara during 2013-2014 showed a higher rate of perinatal mortality of 37.6 per 1000 births, SBR of 23.9 per 1000 births and ENMR of 14.2 per 1000 live births.^8^ The differences could be due to lack of access, lack of resources, lack of awareness, and poor quality of care provided during pregnancy and delivery in the community.

Previous studies has shown male gender had higher risk of dying in perinatal period than female gender.^9,10^ However, there was no gender differences in our study.

More than half of deaths were contributed by stillbirths in this study which was consistent to other studies.^5–6–8^ Hence there is a scope of improvement to reduce stillbirths by providing good antenatal care and counseling to pregnant women as well as antenatal surveillance for high risk pregnancies at all levels of health care. Similarly 5 out of 7 ENND were born preterm in this study. Prematurity contributed significantly to the early neonatal deaths in other studies.^4,5,6,8^

Prevention of preterm labor by good antenatal care and identification of high risk pregnancies, labor monitoring and provision of good neonatal services to preterm neonates including availability of surfactant and respiratory support would further lead to better newborn survival and reduction of perinatal mortality.

Although this study had inherent bias and limitations being a hospital based study, this provides a current status of perinatal deaths at a tertiary care center and provides insights for improvement in future.

## CONCLUSIONS

Stillbirths and prematurity contributed significantly to the perinatal mortality. Provisions for antenatal surveillance and monitoring, timely referral of high risk pregnancies and good neonatal care to preterm neonates are needed to further reduce perinatal deaths.

## Conflict of Interest

**None.**
